# Electric fields enhance Diels–Alderase catalysis in abyssomicin C biosynthesis

**DOI:** 10.1039/d5cc05141j

**Published:** 2026-01-06

**Authors:** Rodrigo Recabarren, Sam T. Johns, H. Adrian Bunzel, Esteban Vöhringer-Martinez, Marc W. van der Kamp

**Affiliations:** a Departamento de Físico-Química, Facultad de Ciencias Químicas, Universidad de Concepción Concepción Chile evohringer@udec.cl; b School of Biochemistry, University of Bristol Bristol BS8 1TD UK marc.vanderkamp@bristol.ac.uk; c Max Planck Institute for Terrestrial Microbiology Marburg Germany

## Abstract

Natural Diels–Alderases catalyse [4+2] cycloadditions by preorganizing substrates into reactive conformations. However, the roles of other catalytic factors, such as electrostatic effects, remain elusive. Here, we combine conceptual density functional theory (CDFT) descriptors and electric field analysis to unravel the electrostatic basis of activity in the Diels–Alderase AbyU. Previously, four different enzyme-substrate poses were identified, of which two showed catalytically favorable free energy barriers based on quantum mechanical/molecular mechanical (QM/MM) reaction simulations. Here, we show that atom-condensed Fukui functions can predict the reactivity from reactant conformations alone, focusing on the diene carbons involved in bond formation. The importance of the enzyme-diene interaction is supported by electric field analysis, which shows how reactivity of enzyme-substrate poses correlates with alignment of the enzyme field along the diene moiety. Our findings establish a basis for predicting and engineering Diels–Alderase activity based on electrostatic and electronic reactivity features.

## Abstract 2

β-Barrel enzymes that promote Diels–Alder reactions, such as AbyU, are promising biocatalysts. Here, we combine atom-condensed Fukui functions and electric field analysis to show that AbyU provides a permanent electric field to catalyse the reaction.

## Graphical abstract

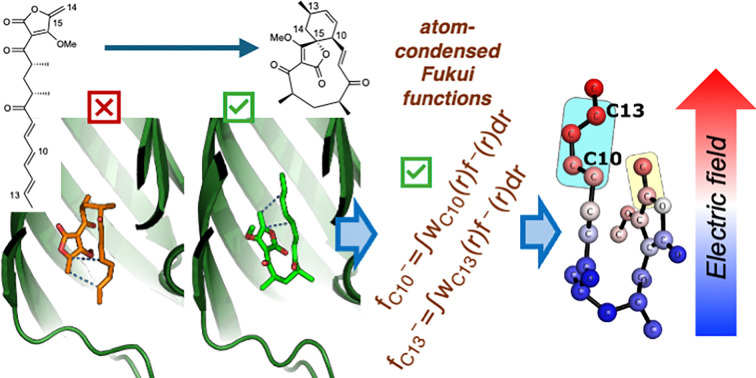

The Diels–Alder reaction is a [4+2] cycloaddition reaction that is widely used in organic synthesis.^1^ It is a powerful tool for the construction of complex cyclic compounds, with synthetic applications in natural products, vitamins, hormones, polymers, drugs, and agrochemicals.^2^ Despite its versatility, achieving high activity and (stereo)selectivity under mild, aqueous conditions remains a challenge—particularly for unactivated substrates. The discovery and development of biocatalysts for the Diels–Alder reaction could address these limitations to promote cycloadditions with high selectivity and efficiency. Recently, several Diels–Alderases have been characterized that are involved in the synthesis of different natural products.^3,4^ These enzymes provide a promising starting point for the development of new biocatalysts, opening up sustainable routes to a wide variety of valuable chemical building blocks.

One recently discovered *bona fide* natural Diels–Alderase is AbyU, catalyzing an intramolecular Diels–Alder reaction involved in the biosynthesis of abyssomicin C, a potent antibiotic.^5^ AbyU is a highly stable β-barrel enzyme^6^ that functions as a standalone pericyclase catalyzing the reaction of an exocyclic methylene group and conjugated diene to form the respective carbocycle (Fig. 1A). One challenge in designing biocatalysts for the Diels–Alder reaction is understanding how the substrate-enzyme interaction affects reactivity. A recent study indicated that a similar Diels–Alderase can use a combination of induced-fit/conformational selection and local electrostatic stabilization to catalyse the reaction.^7^

**Fig. 1:**
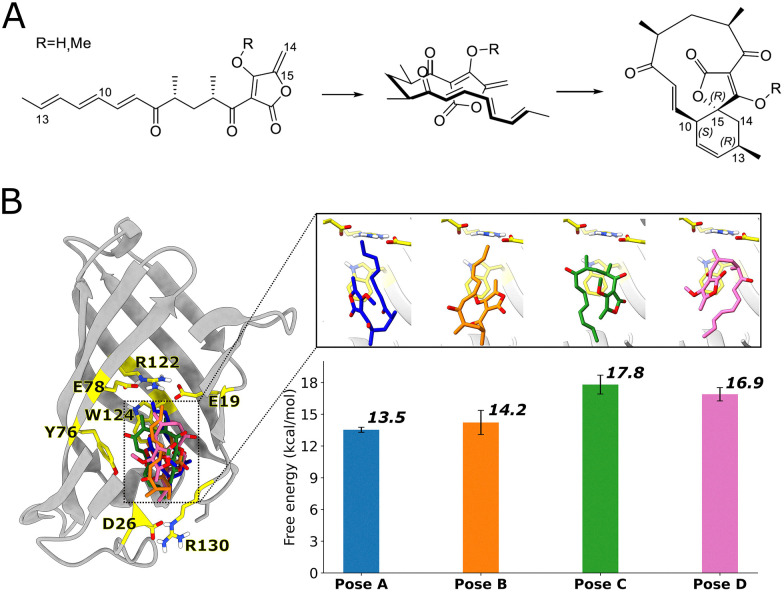
(A) Reaction scheme showing initial folding of the AbyU substrate and subsequent cyclization. A methoxy analogue (R=CH_3_; native substrate: R=H) is often used in experiments and simulations.^3,6,8^ C14 and C15 serve as the dienophile in the reaction, while C10 and C13, located at the end of the triene moiety, are the diene atoms involved in bond formation. (B) AbyU structure with the four different substrate poses in the cavity, together with the free energy barriers for each pose as previously calculated using QM/MM umbrella sampling simulations (DFTB2/ff14SB, with corrections to M06-2X/6-31G(d,p)).^8^

For AbyU, we recently identified four main possible binding poses for the substrate in the active site: poses A, B, C, and D (Fig. 1B).^8^ The activation free energies associated with each of these binding poses were estimated by QM/MM molecular dynamics (MD) simulations. Poses A and B, where the substrate is in an “up” conformation (diene and dienophile located towards the middle of the β-barrel), had smaller activation barriers compared to poses C and D (“down” conformation, with diene and dienophile near the end of the β-barrel). Although calculating activation free energies through QM/MM MD reaction simulations is a powerful approach for studying enzymatic catalysis, it remains computationally expensive.^9^ While the computational cost can be reduced by employing semiempirical Hamiltonians, faster methods would be desirable for rapid *in silico* screening of bioreactivity.

In this work, we applied conceptual density functional theory (CDFT)^10^ to predict atom-based reactivities using enzyme-reactant conformations only, as a computationally efficient alternative to full QM/MM MD simulations to assess reactivity in Diels–Alderase AbyU. Global and local CDFT descriptors, such as atom-condensed Fukui functions,^11^ have been shown to correlate with thermodynamic driving forces for some reactions.^12^ Specifically for Diels–Alder reactions, various previous studies provide evidence for a direct relation with kinetic data, such as activation barriers.^13–16^ Interestingly, these descriptors have shown little correlation with thermodynamic quantities in Diels–Alder reactions,^13^ suggesting that they capture instead reactivity features associated with reaching the transition state. While they have traditionally been used to describe reactivity in gas-phase and aqueous environments—often employing implicit solvent models—the recently proposed Boltzmann-weighted atom-condensed Fukui function approach has extended their application to enzymatic reactions.^17^ Here, we use this approach to analyse the reactivity of the possible enzyme-substrate poses in AbyU. Furthermore, we analyse the electric field exerted by the enzyme environment on the substrate to explain the reactivity of poses depending on their orientation. Both approaches are promising and complementary methods for the study of substrate reactivity in β-barrel Diels–Alderases.

To test if atom-condensed Fukui functions (as well as bond-forming distances and HOMO–LUMO gaps) are able to predict reactivity in AbyU catalyzed Diels–Alder reactions, we performed QM/MM molecular dynamics simulations of the reactant state for each of the four identified substrate poses (Note S1). Two replicas were used for each pose. The initial structures for each replica came from a previous study that used umbrella sampling simulations to bring the system from the product to the reactant state.^8^ Previous computational studies have shown that the substrate reacts through an asynchronous Diels–Alder reaction with a normal electronic demand, where the diene fragment acts as the nucleophile and the dienophile fragment as the electrophile.^5^ To quantify their reactivity, we calculated Fukui functions for both fragments. To this end, the substrate was divided into two fragments (see Fig. S1). For the diene fragment, atom-condensed nucleophilic Fukui functions (f^−^) were calculated, whereas electrophilic Fukui functions (f^+^) were calculated for the dienophile fragment (Note S2). For each pose, two independent QM/MM MD simulations (replicas) of 100 ps were performed using the AmberTools module sander at the DFTB2^18^/Amber-ff14SB level of theory. The Fukui functions were estimated for 50 equally spaced frames from each replica, using the frontier molecular orbital approximation, as implemented in the ChemTools program.^19^ Electron densities for each frame were obtained from single-point QM/MM calculations using the Gaussian/Amber interface^20^ at the B3LYP/6-31G**/ff14SB level of theory. Electric fields and electrostatic potentials were analyzed using TUPA^21^ and FieldTools^27^ based on QM/MM MD frames (200 per replica; Note S3).

The four poses sample similar bond-forming distances and show no significant difference in HOMO–LUMO gaps calculated in the enzyme environment (Fig. S4). Thus, standard conformational selection or global electronic reactivity measures do not explain the difference in reactivity between the poses here. In contrast, the averaged atom-condensed nucleophilic Fukui function f^−^ for the atoms of the diene fragment directly involved in the Diels–Alder reaction (C10 and C13) does show a clear trend towards larger values for both carbons in poses A and B (Fig. 2), *i.e.*, greater reactivity and nucleophilic character. In poses C and D, the descriptors predict a lower reactivity for both carbons. These reactivity predictions based on f^−^ are in agreement with the QM/MM activation free energies shown in Fig. 1, where poses A and B in an “up” conformation exhibited increased reactivity, reflected in lower barriers. To test the effect of the enzyme environment, we performed calculations on the same substrate conformations in vacuum or using implicit solvent (Fig. 2 and Fig. S2), which indicated no significant difference in the average f^−^ values between the poses. This demonstrates that the differences in reactivity between poses A, B and C, D are not due to internal conformational or electronic differences, but rather due to the enzyme environment catalysing the reaction in poses A and B.

**Fig. 2:**
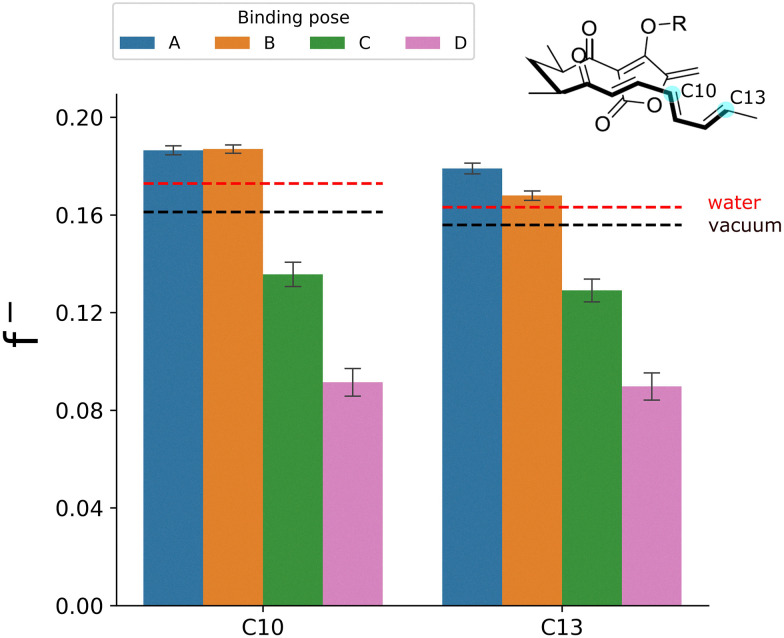
Boltzmann-weighted atom-condensed nucleophilic Fukui functions (f^−^) of diene carbons C10 and C13 (see inset) in all four enzyme-substrate poses (based on 100 frames each). Error bars represent the standard error of the mean. Differences are statistically significant for all pairs (*p* < 0.05 in two-sample *t*-tests), except for atom C10 between poses A and B (*p* = 0.83). The dotted horizontal lines represent the average f^−^ values for all poses in aqueous medium (using an implicit solvent model) and in vacuum. In the latter two cases, only average values for all poses are represented, since the variations between poses are minimal (see Fig. S2).

We also calculated the atom-condensed f^+^ functions for the dienophile fragment (atoms C14 and C15, see Fig. S3). Unlike the nucleophilic Fukui function, the f^+^ function is not localized on the dienophile atoms; instead, it is distributed across the tetronic acid ring (see Fig. S4). This broader distribution may reduce its effectiveness in predicting electrophilic reactivity. Although the differences between the poses are statistically significant (*p* < 0.05), they are much smaller (>3*x*) than for f^−^ and do not align with the expected reactivity. Together, these results suggest that primarily the reactivity of the diene fragment is affected by electrostatic modulation of the enzyme, and this can account for the reactivity difference between poses A, B and C, D.

Our results indicate that the enzyme not only catalyzes the reaction by accommodating the substrate in a folded or ‘pre-organized’ conformation due to complementarity with the active site,^5,22^ with the diene and dienophile in close proximity (Fig. S4, panel B), but also applies an electric field that favours the reaction. Furthermore, the reactivity predicted by atom-condensed f^−^ in the aqueous environment is lower than the reactivity for poses A and B (as expected), but higher than for poses C and D. This is also consistent with activation free energies calculated in explicit solvent using QM/MM umbrella sampling (Note S4 and Fig. S5), validating the use of atom-condensed Fukui functions for predicting reactivity in different environments.

To rationalize the differences in reactivity observed for each pose, and to understand how the enzyme polarizes the substrate, we calculated the electrostatic potential of the enzyme environment at individual substrate atoms (Fig. 3A and Note S3). This potential indicates that the enzyme generates an electric field that is predominantly directed from the bottom of the cavity (blue shaded atoms in Fig. 3A) towards the top (red shaded atoms). The result of this is that the diene portion in poses A and B becomes more polarized, with C10 becoming more electron-rich and C13 more electron-poor (see Fig. S6), enhancing Diels–Alder reactivity.

**Fig. 3:**
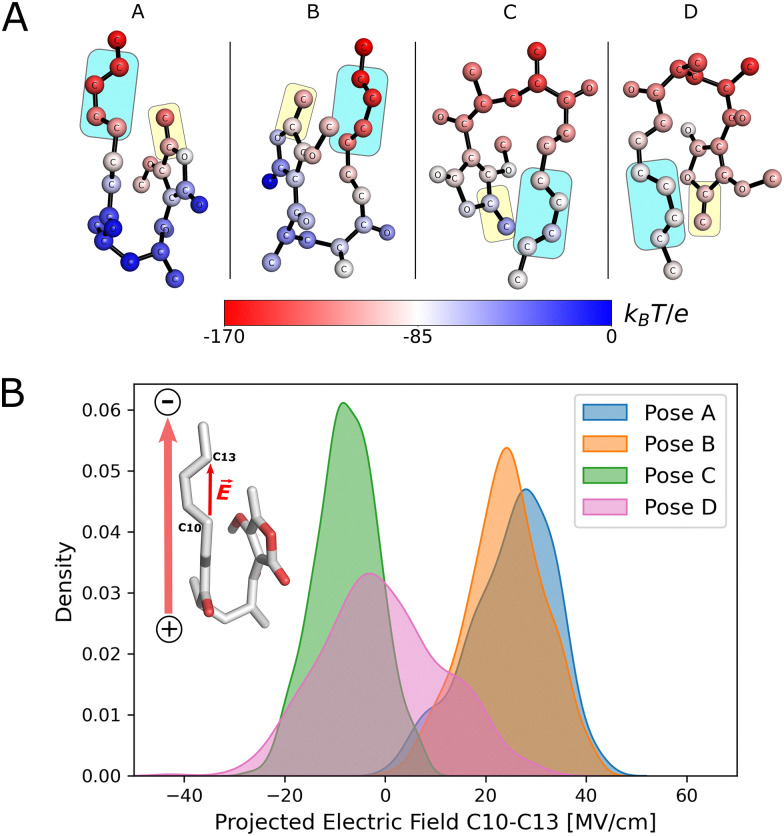
(A) Enzyme electrostatic potential calculated at substrate atoms, averaged over 400 configurations of each pose. The diene is indicated with a pale blue rectangle, the dienophile with a pale yellow rectangle. Atom colouring indicates the electrostatic potential at the atom position according to the scale bar. Changes in (Hirshfeld-I) atomic charges of the diene due to the enzyme environment are indicated in Fig. S6. (B) Kernel density estimation of the enzyme electric field projected on the C10 → C13 vector based on 400 configurations of each pose.

The ‘bottom-to-top’ enzyme electric field vector is approximately aligned along the diene fragment for all poses. We thus analysed the projection of the electric field onto a vector defined from C10 to C13, which approximately resembles the orientation of the diene (Fig. 3B). For poses A and B, the projected electric fields have positive values for all substrate conformations and their distributions are similar. In contrast, in poses C and D, where the substrate is in an inverted conformation relative to A and B, the values are predominantly negative (especially for pose C) and lower in absolute value. Negative values imply that the electric field is aligned in the opposite direction of the C10–C13 vector.

We also studied the enzyme electric field projected onto vectors based on the atoms that form the new carbon–carbon bonds in the reaction (vectors C13 → C14 and C10 → C15), as it was shown that an electric field applied in the direction of the reaction axis (bonds to be formed) in a Diels–Alder reaction can decrease the activation barrier.^23–25^ The resulting electric field distributions show no clear trend with the expected reactivity of the poses, suggesting that the enzyme is not exerting an electric field in this orientation to enhance catalysis (see Fig. S7).

For pose A, the electric field is very well aligned with the vector defined by atoms C10 and C13 (C10 → C13) (Fig. S8). For pose B, the alignment is not as optimal, but the electric field is still predominantly oriented in that direction (probability peak >50%). In the case of poses C and D, the alignment is predominantly in the opposite direction (negative values) and is generally poor. Pose C has a higher degree of alignment in the opposite direction to the defined vector, in agreement with the values of the projected electric field (Fig. 3B). Therefore, the degree of positive alignment of the enzyme electric field with the diene correctly predicts the order of reactivity calculated by QM/MM umbrella sampling simulations.

To analyze the origin of the catalytic electric field provided by the enzyme, the field projected on the C10 → C13 vector was decomposed into residue contributions (Fig. S9). This reveals that the charged amino acid residues that seal the cavity in the β-barrel (E19, R122, E78 at the ‘top’ of the active site, see Fig. 1) contribute the most to the electric field in poses A and B. For poses C and D, charged residues D26 (part of the capping loop) and R130, which form a salt bridge at the bottom of the cavity, are the primary contributors to the electric field. For all four poses, the diene moiety is positioned close to the charged residues that contribute most significantly to the projected field.

Overall, the analysis of the enzyme electric field allowed us to rationalize the differential diene reactivity of enzyme-substrate poses indicated by the atom-condensed Fukui functions, and thus explain the differences in the previously calculated activation free energies. In poses A and B, the electric field is oriented such that it generates a favourable polarization of the HOMO in the diene fragment (see Fig. 3A and Fig. S6), which makes it more reactive (as predicted by the Fukui functions). In the case of poses C and D, the electric field is predominantly in the opposite direction (and not well oriented), thus decreasing the diene reactivity. Importantly, our work shows that for β-barrel Diels–Alderases, a computationally inexpensive analysis of the effect of the enzyme environment is sufficient to predict the relative reactivity of enzyme-substrate poses.

β-Barrel Diels–Alderases have the promise to serve as effective biocatalysts in sustainable routes to synthesise a wide variety of complex and valuable chemical compounds.^3,6,26^ Here, we demonstrate that the electronic properties governing enzyme activity, relevant for engineering such Diels–Alderases, are obtained by efficient computational analysis of alternative substrate poses. For the Diels–Alderase AbyU, atom-condensed Fukui functions correctly predict the reactivity of substrate poses, avoiding the need for full QM/MM free energy calculations of the reaction. Furthermore, electric field analysis then reveals the source of the enzyme-induced reactivity: specific sets of charged residues that provide an electric field aligned to the diene moiety. For the β-barrel Diels–Alderases, the reaction-promoting effects can thus be identified efficiently, based on enzyme-substrate poses only. This is likely related to the fact that the enzyme holds the substrate in a pre-TS (‘folded’) conformation, and that the catalytic effects for the reaction can already be quantified without considering the (not highly polarized) enzyme-bound transition state. (For other enzymes, analysis on transition states is likely required, especially if these are more highly polarized.^27,28^) We anticipate that our approach will allow to quickly identify reactive poses and pinpoint mutation hotspots for redesign,^22^*e.g.* to increase the strength and alignment of the electric field along the substrate diene.

RR – investigation, visualisation, formal analysis, writing: original draft & review and editing, funding acquisition. STJ – investigation, writing: review and editing. AB – investigation, visualization, writing: review and editing. EVM – conceptualization, methodology, writing: review and editing, supervision, funding acquisition. MWvdK – conceptualization, formal analysis, writing: original draft & review and editing, supervision, data curation, funding acquisition.

## Conflicts of interest

There are no conflicts to declare.

## Supplementary Material

CC-062-D5CC05141J-s001

## Data Availability

Data for this article, including MD trajectories, topology files, input and output files of atom-condensed Fukui descriptors and electric field calculations are available at Zenodo at https://doi.org/10.5281/zenodo.16995566. Supplementary information is available. See DOI: https://doi.org/10.1039/d5cc05141j.
