# Supplementary material for: Electric fields enhance Diels–Alderase catalysis in abyssomicin C biosynthesis
Source: Chem Commun (Camb). 2026 Jan 6;62(8):2594–8. doi: 10.1039/d5cc05141j (PMC12772626; doi:10.1039/d5cc05141j)
Supplement: CC-062-D5CC05141J-s001 [file CC-062-D5CC05141J-s001.pdf]

Supplementary Information (SI) for ChemComm.
This journal is © The Royal Society of Chemistry 2025

Supplementary Information

Electric fields enhance Diels–Alderase catalysis in Abyssomicin C biosynthesis

Rodrigo Recabarren[a], Sam T. Johns[b], H. Adrian Bunzel[c], Esteban Vöhringer-Martinez*[a] and Marc W. van der Kamp*[b]

[a] Departamento de Físico-Química, Facultad de Ciencias Químicas, Universidad de Concepción, Concepción, Chile
[b] School of Biochemistry, University of Bristol, Bristol BS8 1TD, UK
[c] Max Planck Institute for Terrestrial Microbiology, Marburg, Germany

email: Marc.VanderKamp@bristol.ac.uk; evohringer@udec.cl

Note S1. System preparation and QM/MM MD Simulations

Details of system preparation, docking calculations, and umbrella sampling simulations that generated the four binding poses studied here are reported in our previous work,[1] with the protein structure based on PDB ID 5DYV, chain A, and a TIP3P solvent sphere (20 Å radius) around the substrate. The QM/MM MD simulation protocol was also adopted from that previous study, including the use of the SCC-DFTB/ff14SB level of theory with a 1 fs time step, and a QM region consisting of the substrate only. General Amber Force Field atom types were assigned for the calculation of Van der Waals interactions. For each pose, two different enzyme-reactant starting structures were obtained for simulation, taken from the reactant minima of two individual series of QM/MM MD umbrella sampling simulations of the reaction. These represent the same binding poses initially identified with docking. For each reactant structure (2 per pose), a 100 ps QM/MM MD simulation was performed under NVT conditions at 300 K, considering two replicas. As in the previous work, all atoms beyond a 20 Å cutoff were restrained using the *ibelly* option in sander. Non-bonded interactions were calculated with a 10 Å cutoff. All simulations described were carried out using the *sander* module of the AmberTools18 software package.[2]

Note S2. Atom-condensed Fukui function calculations

From the 100 ps trajectories, with two replicas for each binding pose, 50 evenly spaced frames were extracted from each trajectory. QM/MM single-point calculations were performed at the B3LYP/6-31G**/ff14SB level of theory using the sander/Gaussian interface,[3,4] with a 10 Å cutoff applied for non-bonded MM and QM/MM interactions. The QM region was reduced to include only the diene and dienophile fragments (see Fig. S1) for the subsequent calculation of nucleophilic (f^-) and electrophilic (f^+) Fukui functions.

Molecular electron densities stored in the checkpoint files from the QM/MM calculations in Gaussian16[4] were processed using ChemTools[5] to compute atom-condensed Fukui functions. The f^+ and f^- indices were calculated using the frontier molecular orbital (FMO) approximation, where the electron densities of the lowest unoccupied molecular orbital (LUMO) and the highest occupied molecular orbital (HOMO) were used. For a system of N_0 electrons, they are defined as follows:

$$f_{N_0}^{+} = \rho_{N_0+1} - \rho_{N_0} \approx \rho^{LUMO}(r)$$

$$f_{N_0}^{-} = \rho_{N_0} - \rho_{N_0-1} \approx \rho^{HOMO}(r)$$

The Fukui function was condensed to individual atoms using the Hirshfeld-I partitioning method[6] in combination with the fragment of molecular response (FMR) approach. This methodological combination has proven to accurately capture the reactivity of α,β-unsaturated systems.[7] The weight function used to partition the electron density in the Hirshfeld-I approach, associated to atom A, is defined as:

$$w_A^{HI}(r) = \frac{\rho_A^0(r)}{\sum_{A=1}^{M} \rho_A^0(r)}$$

where $\rho_A^0(r)$ represents the spherically averaged proatomic electronic density of atom A, calculated as a linear combination of proatomic densities with integer populations that are closest to the actual population of the atom in the molecule. This is achieved through an iterative self-consistent procedure. Finally, the condensed Fukui function for atom A in the molecule is obtained using the Hirshfeld-I (HI) weight function as:

$$f_A^{\pm} = \int w_A(r) f^{\pm}(r) dr$$

Boltzmann-weighted atom-condensed Fukui functions[8] were then calculated using the 50 frames extracted from each trajectory, averaging the $\boldsymbol{f_A^{\pm}}$ values for each configuration (100 configurations for each binding pose). Since an electronic embedding scheme was employed in the B3LYP/6-31G**/ff14SB QM/MM calculations, the polarized electron density at fixed atomic positions yields atom-condensed Fukui function values that capture the influence of the molecular environment on the fragments considered.

Note S3. Electrostatic potential and electric field analysis

The electrostatic potential exerted by the enzyme and surrounding water molecules at each of the substrate atoms was calculated using the classical Coulomb expression:

$$\boldsymbol{V_E(r) = \frac{1}{4\pi\varepsilon_0} \sum_i \frac{q_i}{|r - r_i|}}$$

Where *r* denotes the point at which the potential is evaluated (substrate atoms), r_i is the position of a nonzero charge q_i (MM partial charge) and $\boldsymbol{\varepsilon_0}$ is the vacuum permittivity. This was performed using a modified version of FieldTools (https://github.com/bunzela/FieldTools).

Electrostatic potentials and electric fields ($\vec{\boldsymbol{E}}$) were calculated for 200 frames per trajectory (each 0.5 ps), resulting in 400 frames for each binding pose.

Electric field calculations were performed with the program TUPA.[9] The "BOND" mode was chosen to calculate projected electric fields on different bond vectors. Here, the midpoint of 2 selected atoms is tracked and is used as a probe to calculate $\vec{\boldsymbol{E}}$. The projection of $\vec{\boldsymbol{E}}$ onto the defined bond vector is calculated as:

$$\boldsymbol{E_{proj}} = \frac{\vec{\boldsymbol{E}}_{\boldsymbol{total}} \cdot \vec{\boldsymbol{r}}_{\boldsymbol{bond}}}{|\vec{\boldsymbol{r}}_{\boldsymbol{bond}}|}$$

The alignment percentage to the defined bond vector is calculated as:

$$\%alignment = \frac{E_{proj}\ x\ 100}{\left|\vec{E}_{total}\right|}$$

All partial charges of the system (i.e. partial charges from all atoms in Note S1, protein and solvent) were considered in the calculations, except those of the substrate.

Note S4. Solvent-only QM/MM umbrella sampling simulations

The free energy barrier for the reaction in solution was calculated using umbrella sampling along the same reaction coordinate optimized for the enzymatic reaction as reported previously.[1] The same QM method was used for the substrate: SCC-DFTB. All simulations were performed in a rectangular periodic box, both for umbrella sampling and the generation of initial structures.

The system was prepared by solvating the product structure in a periodic box of TIP3P water, with a box buffer (minimal distance between solute and edge of the box) of 20 Å. Topology and coordinate files were generated using previously generated GAFF parameters for the product.[1] The system was equilibrated using molecular mechanics (MM) with the Amber pmemd.MPI module under periodic boundary conditions. The default direct-space non-bonded interaction cutoff of 10 Å was used, and the Particle Mesh Ewald (PME) method was applied for long-range electrostatics. The equilibration protocol included:

1. *Minimization of solvent and solute hydrogen positions.*
   300 steps of minimization with restraints on solute heavy atoms (force constant: 100 kcal·mol^{-1}·Å^{-2}).
2. *Solvent heating and equilibration.*
   Random velocities assigned at 50 K, followed by 50 ps NPT heating to 300 K with a 2 fs timestep using SHAKE constraints. Langevin dynamics with a collision frequency of 2 ps^{-1} and the Berendsen barostat (pressure relaxation time: 1 ps) were considered. Solute atoms were restrained with a force constant of 25 kcal·mol^{-1}·Å^{-2}.
3. *Minimization and quick heating of the full system.*
   300 additional minimization steps, followed by heating to 298 K over 20 ps in the NVT ensemble, using a 2 fs timestep, SHAKE, Langevin dynamics (collision frequency: 1 ps^{-1}), and random velocities assigned at 25 K.
4. *Final equilibration.*
   600 ps of NPT equilibration with Langevin dynamics using the Berendsen barostat.

Ten snapshots were extracted at regular intervals from the final 200 ps of equilibration to initiate QM/MM (SCC-DFTB/TIP3P) umbrella sampling simulations. The umbrella sampling protocol followed that used for the enzymatic system,[1] with the sole difference being that all simulations were performed in the NPT ensemble, using the same parameters as the equilibration stage.

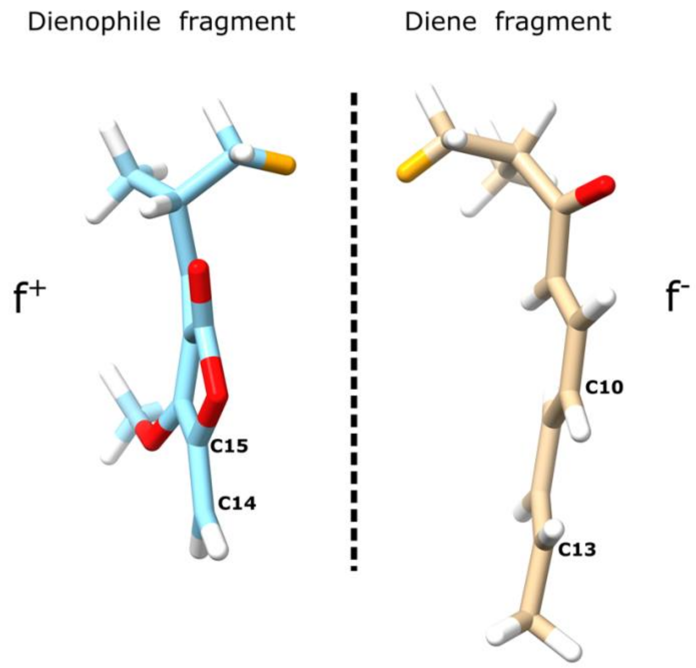

Figure S1. Fragments considered for the single-point QM/MM calculations to compute nucleophilic and electrophilic atom-condensed Fukui functions. Link atoms are highlighted in orange.

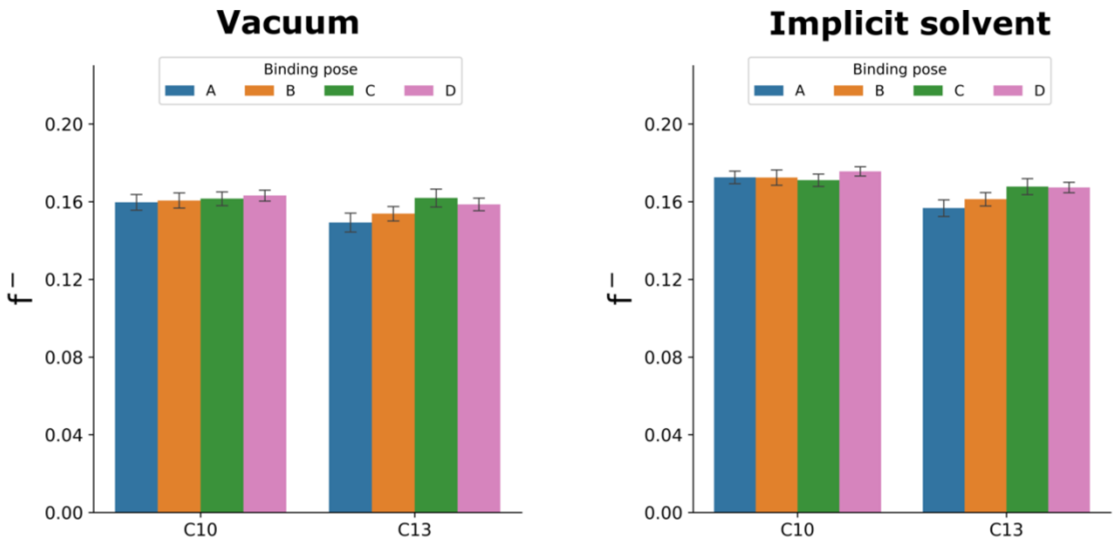

Figure S2. Atom-condensed nucleophilic Fukui functions (f^{-}) of diene carbons C10 and C13 in all four enzyme-substrate poses, both in vacuum and in aqueous medium (using the default Polarizable Continuum Model implemented in Gaussian16). Error bars represent the standard error of the mean. Differences are not significant, based on $p > 0.05$ for two-sample t-tests on all pairwise comparisons of atoms C10 and C13 in the four poses, apart from $p=0.043$ for C13 in poses A and D with implicit solvent.

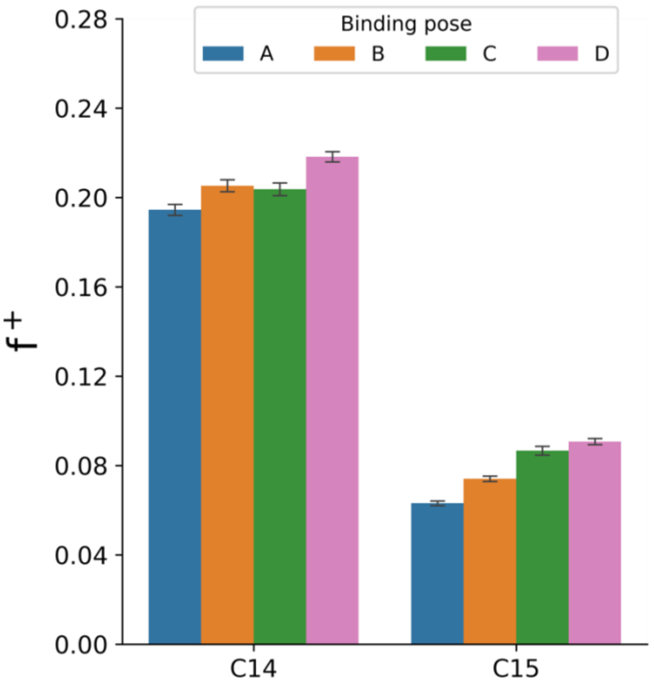

Figure S3. Atom-condensed electrophilic Fukui functions (f^+) of dienophile carbons C14 and C15 in all four enzyme-substrate poses. Error bars represent the standard error of the mean.

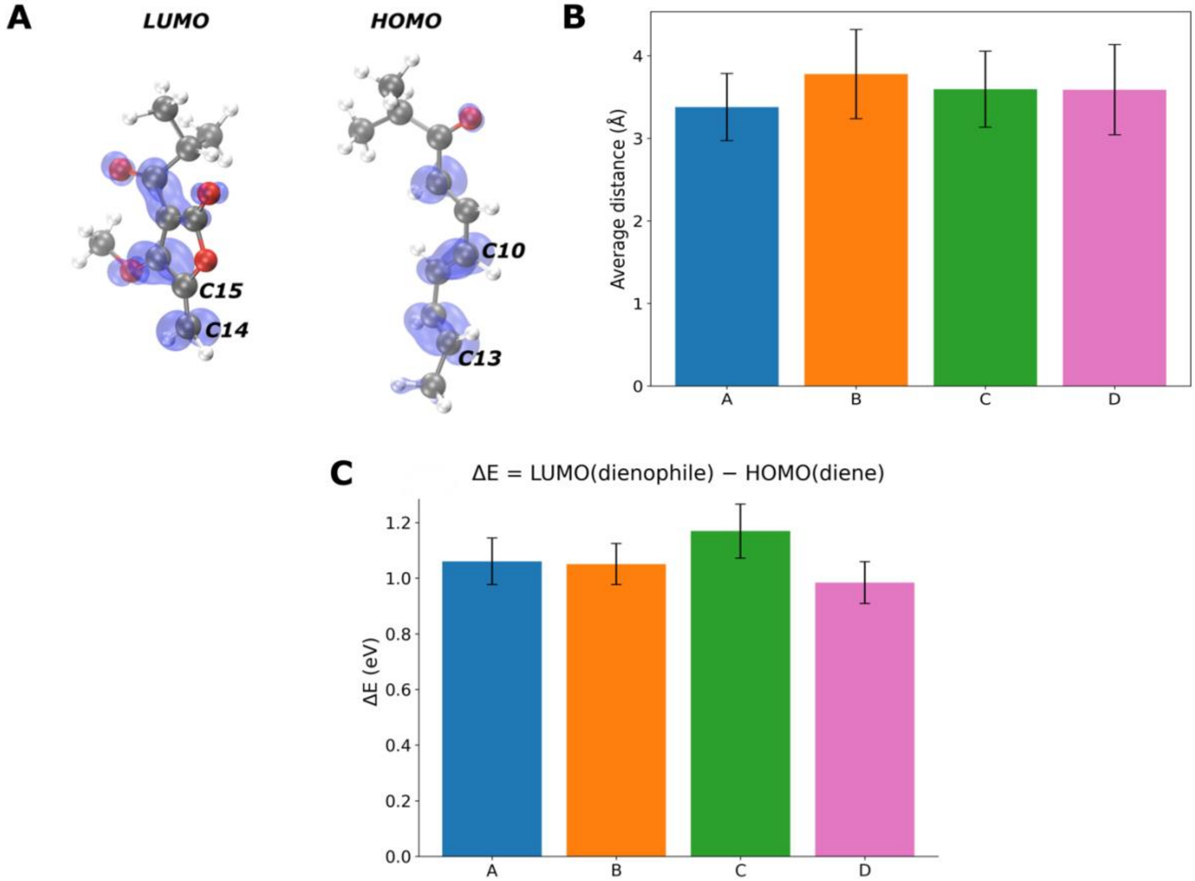

Figure S4. (A) The f⁺ and f⁻ functions are represented by the frontier molecular orbitals LUMO and HOMO, respectively, for a representative frame of the dienophile and diene fragments of pose A. An isovalue of 0.004 was used. The plot was generated with ChemTools. (B) Average bond-forming distances between the C10–C15 and C13–C14 pairs for the four poses studied (400 frames each). (C) HOMO–LUMO gap calculated for all frames used in the atom-condensed Fukui calculations, averaged for each pose. For B and C, error bars correspond to the standard error from 400 frames.

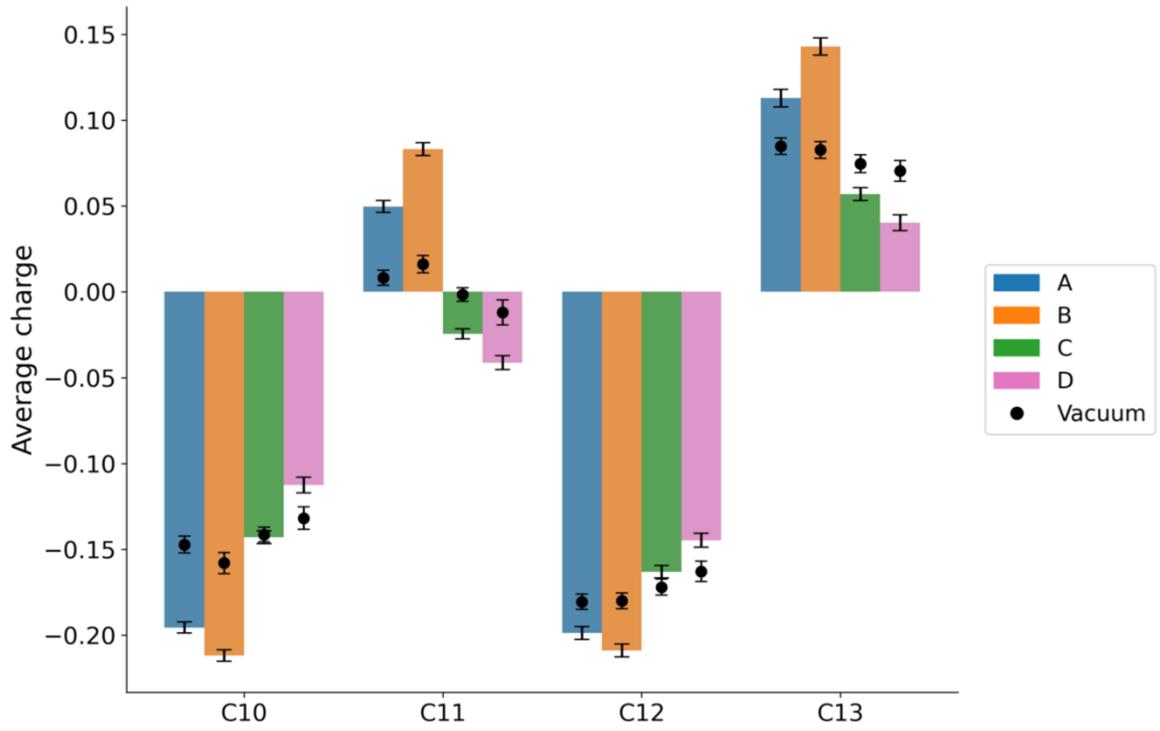

Figure S5. Average atomic charges for carbons C10–C13 of the diene in the four poses (A–D) under the influence of the enzyme (colored bars) and in vacuum (black circular markers). Error bars correspond to the standard error (from 400 conformations each). Across poses A and B, the enzyme environment induces a consistent increased polarization pattern along the C10–C13 conjugated segment, whereas poses C and D exhibit the opposite trend (reduced polarization).

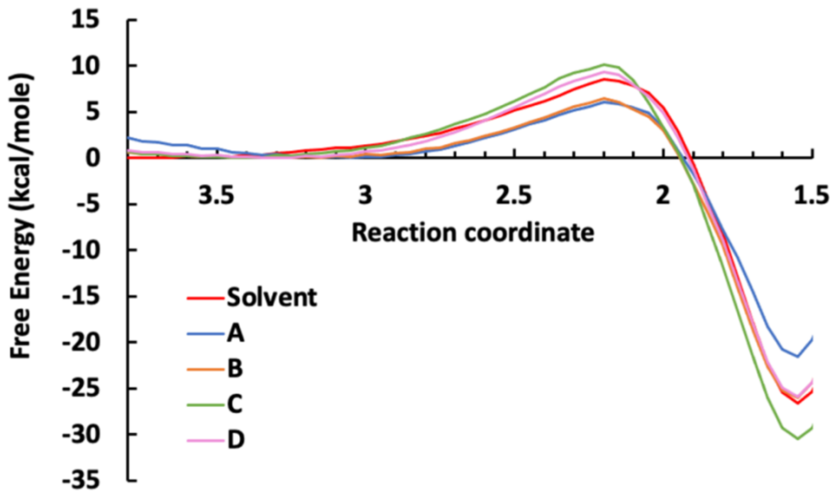

Figure S6. Free energy profiles of the Diels-Alder reaction in AbyU obtained by umbrella sampling simulations for the four binding poses at the DFTB2/ff14SB level of theory, as reported in our previous work,[1] together with the same reaction in aqueous solution (DFTB2/TIP3P level). Note that no correction to M06-2X/6-31G(d,p) QM level is included here, so values differ from those shown in Fig. 1. The energy cost to reach the transition state from reaction coordinate value 3.05 Å (reactant minimum for pose A) is as follows: 6.13 and 6.29 kcal·mol^{-1} for poses A and B, 7.38 kcal·mol^{-1} for water, 9.29 and 8.92 kcal·mol^{-1} for poses C and D. These values are approximately consistent with f^{-} values calculated in implicit solvent being more similar to those for poses A-B than poses C-D (Fig. 2, Fig. S2).

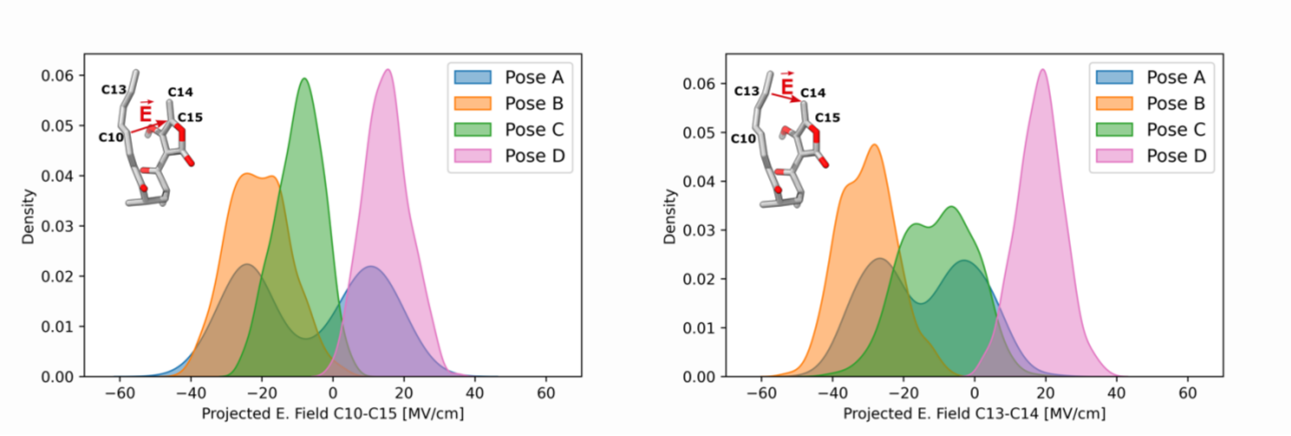

Figure S7. Kernel density estimation of the electric field projected on the C10→C15 and C13→C14 bond vectors.

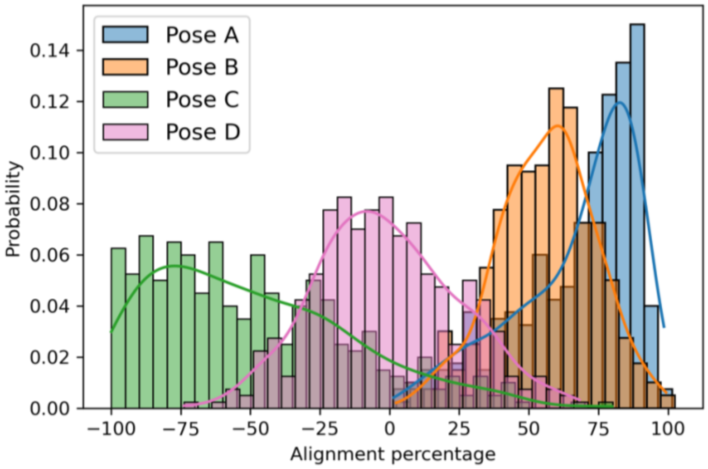

Figure S8. Probability distributions of the signed alignment percentages of the electric field with respect to the defined C10→C13 vector.

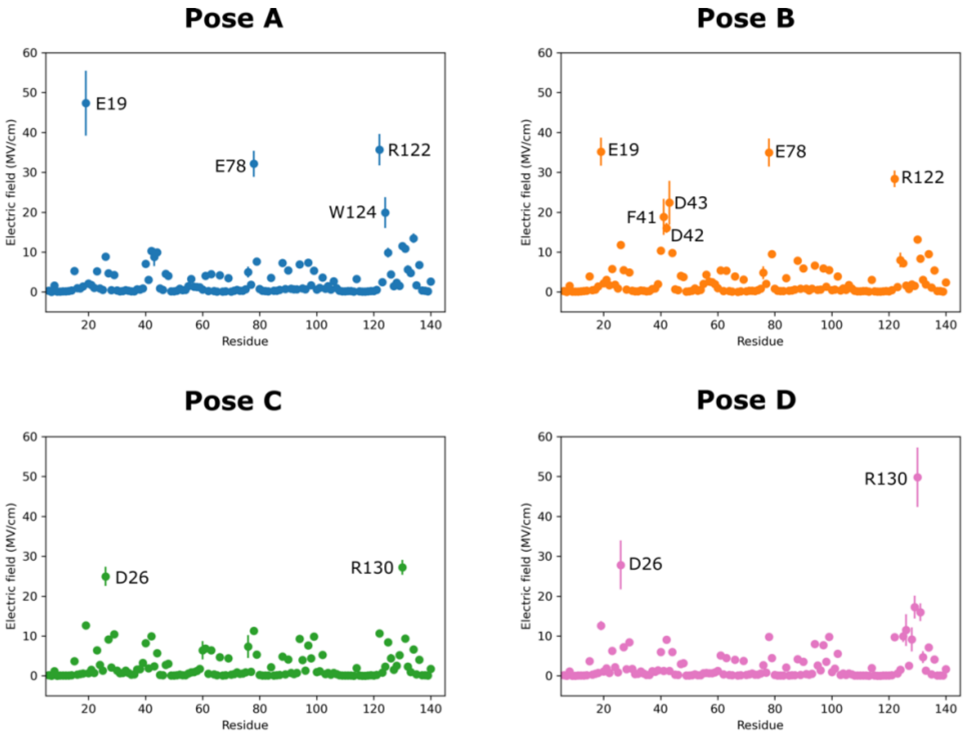

Figure S9. Average contributions for each residue to the projected electric field along the C10→C13 bond vector for each binding pose. Error bars indicate standard deviations calculated from 400 configurations per pose. Residues E19, E78, and R122, which cap the substrate cavity, contribute most to the electric field for poses A and B. For pose A, other residues also contribute significantly, such as W124 (in the substrate cavity close to the diene moiety, see Fig. 1 in the main text). For pose B, residues D42, D43, and F41 also contribute significantly to the projected field. In the case of poses C and D, the primary contributions originate from the charged residues D26 and R130.

References

1 L. Maschio, C. R. Back, J. Alnawah, J. I. Bowen, S. T. Johns, S. Z. Mbatha, L.-C. Han, N. R. Lees, K. Zorn, J. E. M. Stach, M. A. Hayes, M. W. van der Kamp, C. R. Pudney, S. G. Burston, C. L. Willis and P. R. Race, *Chem. Sci.*, 2024, **15**, 11572–11583.

2 D. A. Case, I. Y. Ben-Shalom, S. R. Brozell, D. S. Cerutti, T. E. Cheatham. III, V. W. D. Cruzeiro, T. A. Darden, R. E. Duke, D. Ghoreishi, M. K. Gilson, H. Gohlke, A. W. Goetz, D. Greene, R. Harris, N. Homeyer, Y. Huang, S. Izadi, A. Kovalenko, T. Kurtzman, T. S. Lee, S. LeGrand, P. Li, C. Lin, J. Liu, T. Luchko, R. Luo, D. J. Mermelstein, K. M. Merz, Y. Miao, G. Monard, C. Nguyen, H. Nguyen, I. Omelyan, A. Onufriev, F. Pan, R. Qi, D. R. Roe, A. Roitberg, C. Sagui, S. Schott-Verdugo, J. Shen, C. L. Simmerling, J. Smith, R. Salomon-Ferrer, J. Swails, R. C. Walker, J. Wang, H. Wei, R. M. Wolf, X. Wu, L. Xiao, D. M. York and P. A. Kollman, AMBER 2018 2018.

3 A. W. Götz, M. A. Clark and R. C. Walker, *Journal of Computational Chemistry*, 2014, **35**, 95–108.

4 Gaussian 16, Revision B.01, M. J. Frisch, G. W. Trucks, H. B. Schlegel, G. E. Scuseria, M. A. Robb, J. R. Cheeseman, G. Scalmani, V. Barone, G. A. Petersson, H. Nakatsuji, X. Li, M. Caricato, A. V. Marenich, J. Bloino, B. G. Janesko, R. Gomperts, B. Mennucci, H. P. Hratchian, J. V. Ortiz, A. F. Izmaylov, J. L. Sonnenberg, D. Williams-Young, F. Ding, F. Lipparini, F. Egidi, J. Goings, B. Peng, A. Petrone, T. Henderson, D. Ranasinghe, V. G. Zakrzewski, J. Gao, N. Rega, G. Zheng, W. Liang, M. Hada, M. Ehara, K. Toyota, R. Fukuda, J. Hasegawa, M. Ishida, T. Nakajima, Y. Honda, O. Kitao, H. Nakai, T. Vreven, K. Throssell, J. A. Montgomery, Jr., J. E. Peralta, F. Ogliaro, M. J. Bearpark, J. J. Heyd, E. N. Brothers, K. N. Kudin, V. N. Staroverov, T. A. Keith, R. Kobayashi, J. Normand, K. Raghavachari, A. P. Rendell, J. C. Burant, S. S. Iyengar, J. Tomasi, M. Cossi, J. M. Millam, M. Klene, C. Adamo, R. Cammi, J. W. Ochterski, R. L. Martin, K. Morokuma, O. Farkas, J. B. Foresman, and D. J. Fox, Gaussian, Inc., Wallingford CT, 2016.

5 F. Heidar-Zadeh, M. Richer, S. Fias, R. A. Miranda-Quintana, M. Chan, M. Franco-Pérez, C. E. González-Espinoza, T. D. Kim, C. Lanssens, A. H. G. Patel, X. D. Yang, E. Vöhringer-Martinez, C. Cárdenas, T. Verstraelen and P. W. Ayers, *Chemical Physics Letters*, 2016, **660**, 307–312.

6 P. Bultinck, S. Fias, C. Van Alsenoy, P. W. Ayers and R. Carbó-Dorca, *The Journal of Chemical Physics*, 2007, **127**, 034102.

7 J. Oller, P. Pérez, P. W. Ayers and E. Vöhringer-Martinez, *International Journal of Quantum Chemistry*, 2018, **118**, e25706.

8 J. Oller, D. A. Saez and E. Vöhringer-Martinez, *J. Phys. Chem. A*, 2020, **124**, 849–857.

9 M. D. Polêto and J. A. Lemkul, *Journal of Computational Chemistry*, 2022, **43**, 1113–1119.
